# Health professionals’ willingness to share responsibility and strengthen interprofessional collaboration: a cross-sectional survey

**DOI:** 10.1186/s12909-024-06351-9

**Published:** 2025-01-21

**Authors:** Sophie Karoline Brandt, Stefan Essig, Andreas Balthasar

**Affiliations:** 1 Faculty of Health Sciences and Medicine, University of Lucerne, Lucerne, Switzerland; 2 Center for Primary and Community Care, Faculty of Health Sciences and Medicine, University of Lucerne, Lucerne, Switzerland; 3 Interface Policy Studies Research Consulting, Lucerne, Switzerland

**Keywords:** Interprofessional collaboration, Interprofessional education, Responsibility, Decision-making, Willingness, Doctors, Allied health professionals, Healthcare

## Abstract

**Background:**

Doctors’ unwillingness to share responsibility acts as a major barrier to interprofessional collaboration (IPC). Educating both doctors and allied health professionals in taking on or relinquishing responsibility could enhance IPC. Yet there is no evidence that these educational efforts increase IPC willingness. This study aims to (1) compare the willingness to take on or relinquish responsibility for decision-making in patient care and their willingness to strengthen IPC between members of five main health professions, and (2) investigate associations between the willingness to take on or relinquish responsibility and the willingness to strengthen IPC.

**Methods:**

We conducted a cross-sectional survey targeting pharmacists, doctors, medical practice assistants, nurses, and physiotherapists in Switzerland. Group differences were assessed, and associations were examined using multivariable logistic regression analyses.

**Results:**

Overall, 3670 health professionals participated. Members of all allied health professions were highly willing to take on more responsibility for decision-making for patient care (ranging from 74.3% to 86.5%). Medical practice assistants (52.3%) and nurses (46.8%) were highly willing to relinquish responsibility, less so pharmacists (34.2%) and physiotherapists (37.8%); doctors were torn between high (49.2%) and neither high nor low willingness (38%). Members of all professions were highly willing to strengthen IPC (ranging from 76.4 to 91.2%). We found a strong, statistically significant relationship between willingness to take on more responsibility and willingness to strengthen IPC (OR = 5.30, *p* < .001). The relationship between willingness to relinquish responsibility and willingness to strengthen IPC was smaller (OR = 3.30, *p* < .001).

**Conclusion:**

Increasing the health professionals’ willingness to take on responsibility is crucial to strengthen IPC. Increasing the willingness to relinquish responsibility would likely be less effective. Actions required include educational and political efforts to transfer responsibility to allied health professionals and to enable health professionals to decide when it is appropriate to take on or relinquish responsibility. Given the willingness of many health professionals to strengthen IPC, substantial potential in practice is evident.

**Supplementary Information:**

The online version contains supplementary material available at 10.1186/s12909-024-06351-9.

## Background

Interprofessional collaboration (IPC) between health professionals contributes to improved patient outcomes [1], efficiency gains [2] and increased job satisfaction [3–5]. It is defined as the process by which professionals from different health professions work together to positively impact care [6]. In view of the increasing shortage of skilled professionals and the growing complexity of healthcare, IPC is increasingly fostered by interprofessional education (IPE) programs [7–9] in which students from different health professions learn from, with and about each other to foster collaboration and improve quality of care [10]. The significance of IPC for improvements in healthcare is emphasized by Bodenheimer’s ten building blocks of high performing care, describing IPC as essential for team-based care (Block 4) and for the comprehensiveness and coordination of care (Block 9) [11].

Many barriers undermine successful IPC [12–16]. One main barrier is the doctor dilemma, whereby doctors are unwilling to share responsibility because they fear a loss of power, status, professional socialization and decision-making responsibility [17]. The dilemma is that IPE programs aim to decrease hierarchy [17], but increase shared care and shared responsibility among health professionals to enable IPC and improve healthcare for patients [17]. Shared responsibility is important to firstly avoid power differentials and conflicts in the distribution of activities, tasks and responsibilities between doctors and allied health professionals [17, 18]. Second, Holm [19] provided evidence that in many situations, shared responsibility is essential to give allied health professionals and patients final responsibility for treatment choices when required. Despite this aim, in many health systems doctors remain the sole decision-makers in the care of their patients and allied health professions have little responsibility in the decision-making process [6, 9, 20–22]. Further barriers for IPC are, for example, lack of time and skilled professionals, lack of reimbursement policies [15, 23], missing processes, structures and options for communication, lack of management support [20, 24], lack of understanding of each other’s skills and knowledge [12, 16, 25] and doubts of professionals regarding the benefits of IPC for patients, and resistance to change [15, 26].

To overcome the doctor dilemma and, in a next step, to tackle the further, more structural barriers and to effectively implement IPC in practice, we argue that the willingness of health professionals is a prerequisite in two respects: Firstly, professionals need to be willing to strengthen IPC [18, 20, 27]. They should actively seek to improve how health professionals work together. This willingness is vital for initiating reforms that enhance work and communication processes and improve reimbursement of IPC. Without their collective willingness, initiatives driven by policymakers or individual organizations and associations are unlikely to yield substantial advancements in IPC. Secondly, a prerequisite of implementing IPC is the willingness of doctors to share and, at times, relinquish responsibility. The importance of shared responsibility to enable IPC and the difficulties described by the doctor dilemma highlight this prerequisite. We expand on this prerequisite and argue that allied health professionals also need to be willing to take on or relinquish responsibility. Given the current constraints that do not (yet) allow them to take on additional responsibility [6, 22], *willingness* is the most pertinent concept for allied health professionals’ contribution to IPC. Since willingness is defined as consent or readiness to do something and as inclination or preference [28], we draw parallels between the terms ‘willingness’ and the term ‘intention’ as used in Ajzen’s theory of planned behaviour [29]. Ajzen’s theory of planned behaviour assumes that an individual’s intention directly affects their behaviour [29]. The intention is in turn influenced by attitudes, subjective norms, and perceived behavioural control [29]. In previous studies on IPC and IPE, several factors have already been identified that help explain the conditions under which intentions result in behaviour [30–33]. Similar to Ajzen’s theory, we also assume that an individual’s willingness influences their behaviour. Willingness to strengthen IPC and willingness to take on or relinquish responsibility for decision-making in the care of their patients as prerequisites to overcome barriers and effectively implement IPC in practice have so far only been partially investigated. Many studies have shown that doctors are not willing to relinquish responsibility for the care of their patients [6, 15, 17, 19, 34]. Other studies have discussed how young professionals carry out their roles and responsibilities in the healthcare system [22, 35] and in which areas they expand their scope as their professional experience increases [36], or have focused on interprofessional collaboration in the care of specific patient groups such as frail older adults [16, 37]. There is no evidence regarding whether allied health professionals are willing to take on more responsibility or relinquish responsibility for decision-making in patient care. According to Whitehead [17], IPE programs should encourage students to take on their (limited) responsibilities to overcome the doctor dilemma and involve medical students and doctors in IPC. If there is a relationship between willingness to take on or relinquish responsibility and the willingness to strengthen IPC, health professional education can improve IPC by implementing Whitehead’s recommendation to train both doctors and allied health professionals to take on or relinquish responsibility. For this, it is helpful to know whether there is a relationship between willingness to take on or relinquish responsibility and the willingness to strengthen IPC. This relationship, however, has not yet been studied.

We have therefore conducted a cross-sectional survey study aiming (1) to compare the willingness to take on or relinquish responsibility for decision-making in the care of their patients and their willingness to strengthen IPC between members of five main health professions, and (2) to investigate associations between the willingness to take on or relinquish more responsibility and the willingness to strengthen IPC.

## Methods

### Study design, setting and participants

We conducted a large-scale cross-sectional study in Switzerland using a nationwide online survey targeted at health professionals [38, 39]. Data collection took place over 7 months, from January to July 2022.

The participants included pharmacists, doctors, medical practice assistants, nurses, and physiotherapists - the five largest professional groups in healthcare in Switzerland. In the Swiss healthcare system, medical practice assistants handle administrative tasks and selected medical-technical activities related to diagnostics and treatment [40]. With further training, they can also advise patients with chronic illnesses under medical supervision [41, 42].

A non-probabilistic sampling method was used due to challenges in accessing health professionals. Access followed the recommendations by Alvarez and VanBeselaere [43] and was facilitated through diverse partners and media outlets. Partners included professional associations, pharmacy, and practice networks and interprofessional organizations as well as training and research institutions. These partners informed their members about the survey via various means such as newsletters, intranet, member magazines, or direct mail, providing them with the survey link. After approximately four to eight weeks, reminders were dispatched via email from the professional associations and networks and articles about the survey were published in member magazines to maximize participation. Professionals received no monetary incentive for survey completion.

### Measurement

The study employed a structured questionnaire with closed questions developed for the study (Supplementary Table S1, Additional File 1). The survey was based on a preliminary literature review and qualitative exploratory interviews with experts in the practical provision of healthcare and the training of health professionals. Prior to implementation of the survey, pre-tests were administered involving both survey experts and professionals from the professional groups addressed. The think-aloud protocol method [44] was used during pre-tests. Any questions found to be unclear or incomplete were revised. Survey participants self-reported all provided information. The survey covered key sociodemographic characteristics including sex, age, profession, professional experience, type of employment, and region.

We used the following questions to measure willingness to take on or relinquish more professional responsibility and the willingness to strengthen IPC:

- In your current position, how willing are you to take on more professional responsibility in decision-making for the care of your patients?
- In your current position, how willing are you to relinquish more professional responsibility in decision-making for the care of your patients?
- In your current position, how willing are you to strengthen interprofessional collaboration?

Professional responsibility was explained in the questionnaire as follows: ‘This is about your perspective on the topic of professional responsibility. Professionals have responsibility in clinical decision-making for the care of their patients. They decide, for example, which examination and treatment measures are necessary, which nursing services are important, and whether other professionals should be involved. It is *not* about leadership responsibility in a team.’ In this paper we use the term *responsibility* and always refer to professional responsibility in decision-making for patient care.

The provided choices used a 5-point Likert scale ranging from ‘very low’ to ‘very high’ willingness. Given the extensive and difficult-to-reach target group of our survey and previous studies that have shown that single-item measures save time, are easy to answer, reduce data processing costs [45–47] and are suitable for measuring certain constructs (e.g., job satisfaction [48, 49]), we used single-item measures to measure willingness to take on or relinquish more professional responsibility and willingness to strengthen IPC. Participants took an average of 17 min to complete the survey as it encompassed additional questions on different topics not expounded upon in this article. Questionnaire responses were collected using Qualtrics^®^, Version January 2022 (Qualtrics, Provo, UT, USA).

Informed consent was obtained by informing participants on the first page, prior to completing the questionnaire, about the research purpose of the study, the voluntary nature of participation with the option to withdraw at any time, and the confidentiality of the information they provided.

### Statistical methods

The sociodemographic characteristics were considered as independent variables. The professional groups were established as a categorical variable encompassing pharmacists, doctors, medical practice assistants, nurses, and physiotherapists. Age was categorized into three groups (30 and below, 31–45, 46 and above). Professional experience was treated as a continuous variable. The two variables on willingness to take on and relinquish more responsibility for decision-making were also regarded as independent variables and split into three levels (low, neither low nor high, high) to achieve a relevant number of responses per level. The variable on willingness to strengthen IPC was considered as a dependent variable and split into three levels (low, neither low nor high, high) to achieve a relevant number of responses per level. For all three variables, the responses ‘very low’ and ‘low’ were summarized as ‘low’. The responses ‘very high’ and ‘high’ were summarized as ‘high’. Since relatively few participants indicated a very low or low willingness, this recoding prevented random error in the analysis.

Discrete variables were presented with tables showing the distributions of number and percentage. The continuous variable was described by mean and standard deviation (SD). The five professional groups within the study population were compared using cross-tabulations. T-tests and chi-square tests were used to detect differences between the groups.

Due to the reduction of levels, ordered logistic regression analyses were used to assess associations between the willingness to take on and relinquish more responsibility for decision-making and the willingness to strengthen IPC [50, 51]. A confidence interval of 95% was employed, and a *p*-value less than 0.05 was considered statistically significant. The reference category for the calculation of odds ratios (OR) is indicated in the presentation of the ordered logistic regression outcomes. Given the few missing values, imputation was unnecessary. Following crude analysis, the regression model was adjusted first for sociodemographic characteristics (age and sex) in the basic model, and subsequently for additional variables (profession, professional experience, type of employment, and region) in the extended model.

To assess the robustness of our ordered logistic regression results, sensitivity analyses were performed, involving reclassification of ‘neither low nor high’ willingness responses as ‘low’ willingness for the variables relating to the willingness to take on and relinquish more responsibility. Data analysis was carried out using R Statistical Software, Version 4.2.2 [52].

## Results

### Participants

The survey included a total of 5014 participants. After excluding participants who did not work in a health profession (118), those who did not provide their professional background (385), those who belonged to professional groups that were not included in the study (448), or those who did not respond to any of the three main questions on willingness (393), the adjusted sample comprised 3670 participants.

Among all participants, 17.6% were pharmacists, 15.2% were doctors, 18.5% were medical practice assistants, 15.9% were nurses and 32.9% were physiotherapists. Roughly 10% of pharmacists, 3% of doctors in healthcare, 4% of medical practice assistants, 3% of nurses and 10% of physiotherapists practicing in Switzerland took part in the survey [53–56]. While the sample might not be fully representative of the target population due to the specific recruitment approach, the sex and age distribution across all professional groups in the sample closely align with the target populations [55, 57].

All tests for differences between the groups regarding sociodemographic characteristics of the study participants were statistically significant (*p* < .001) (Table 1). Most of the surveyed pharmacists, doctors, nurses, and physiotherapists were aged 46 and above. In contrast, most medical practice assistants were under 30 years old. The surveyed doctors indicated the most professional experience and medical practice assistants the least. On average, slightly fewer than half of the participants worked in urban areas, with the remaining half situated in intermediate or rural regions.

**Table 1:** Sociodemographic characteristics of study population

	Total	Pharmacists	Doctors	Medical practice assistants	Nurses	Physiotherapists	*P*
**Total (%)**	3670 (100.0)	645 (17.6)	557 (15.2)	680 (18.5)	582 (15.9)	1206 (32.9)	
**Sex (%)**							< 0.001
Female	2879 (78.5)	484 (75.0)	252 (45.3)	674 (99.1)	522 (89.7)	947 (78.5)	
Male	779 (21.2)	156 (24.2)	301 (54.1)	5 (0.7)	60 (10.3)	257 (21.3)	
Diverse	11 (0.3)	5 (0.8)	3 (0.5)	1 (0.1)	0 (0.0)	2 (0.2)	
**Age groups (%)**							< 0.001
30 and below	680 (18.6)	66 (10.2)	9 (1.6)	325 (47.9)	94 (16.2)	186 (15.4)	
31–45	1236 (33.7)	234 (36.3)	133 (24.0)	223 (32.8)	162 (27.8)	484 (40.2)	
46 and above	1749 (47.7)	345 (53.5)	413 (74.4)	131 (19.3)	326 (56.0)	534 (44.4)	
**Professional experience (years on average**,** SD)**							< 0.001
Overall	14.82 (6.39)	14.72 (6.38)	17.70 (4.32)	11.83 (7.11)	16.26 (5.64)	14.23 (6.48)	
**Type of employment (%)**							< 0.001
Employed with management responsibility	1097 (36.3)	96 (20.0)	234 (49.0)	224 (46.6)	207 (20.2)	96 (20.0)	
Employed without management responsibility	1044 (34.6)	55 (11.4)	232 (48.5)	209 (43.5)	424 (41.3)	55 (11.4)	
Self-employed with employees	548 (18.1)	307 (63.8)	11 (2.3)	14 (2.9)	128 (12.5)	307 (63.8)	
Self-employed without employees	332 (11.0)	23 (4.8)	1 (0.2)	34 (7.1)	267 (26.0)	23 (4.8)	
**Region of work (%)**							< 0.001
Urban	1403 (46.4)	267 (48.0)	211 (43.7)	200 (41.8)	209 (43.5)	516 (50.2)	
Intermediate	803 (26.5)	163 (29.3)	153 (31.7)	92 (19.2)	129 (26.8)	266 (25.9)	
Rural	820 (27.1)	126 (22.7)	119 (24.6)	187 (39.0)	143 (29.7)	245 (23.9)	

Percentages were calculated from the number of respondents to each question

### Willingness to take on or relinquish more responsibility and to strengthen interprofessional collaboration

Allied health professionals showed a high willingness to take on more responsibility for decision-making for the care of their patients (Table 2). This willingness was particularly high among nurses (86.5%) physiotherapists (83.1%) and pharmacists (80.4%). Slightly fewer, namely 74.3%, medical practice assistants indicated neither a low nor high willingness to take on more responsibility for decision-making.

Regarding willingness to relinquish more responsibility, the differences between the professional groups are distinct. While pharmacists (14.7%) and physiotherapists (18.6%) are less willing to relinquish more responsibility, around half of the surveyed doctors, medical practice assistants, and nurses have a high willingness in this regard.

The participants showed a high willingness to strengthen IPC. All tests for differences between groups were statistically significant (*p* < .001). The willingness was higher in allied health professionals compared to doctors (76.4%) and particularly high among pharmacists (91.2%), nurses (90.1%) and physiotherapists (84.3%).

**Table 2:** Willingness of health professionals to take on or relinquish more responsibility and to strengthen IPC

	Total	Pharmacists	Doctors	Medical practice assistants	Nurses	Physiotherapists	*P*-value
**In your current position**,** how willing are you to take on more professional responsibility in decision-making for the care of your patients? (%)**							< 0.001
Low willingness	91 (3.1)	19 (3.1)	*	33 (5.5)	14 (2.5)	24 (2.1)	
Neither low nor high willingness	466 (15.8)	103 (16.6)	*	121 (20.2)	62 (11.1)	171 (14.8)	
High willingness	2400 (81.2)	500 (80.4)	*	446 (74.3)	485 (86.5)	961 (83.1)	
**In your current position**,** how willing are you to relinquish more professional responsibility in decision-making for the care of your patients? (%)**							< 0.001
Low willingness	499 (14.8)	87 (14.7)	69 (12.8)	57 (10.0)	75 (13.7)	211 (18.6)	
Neither low nor high willingness	1432 (42.4)	302 (51.1)	204 (38.0)	215 (37.7)	217 (39.5)	494 (43.6)	
High willingness	1449 (42.9)	202 (34.2)	264 (49.2)	298 (52.3)	257 (46.8)	428 (37.8)	
**In your current position**,** how willing are you to strengthen interprofessional collaboration (%)**							< 0.001
Low willingness	84 (2.3)	6 (0.9)	27 (4.9)	12 (1.8)	11 (1.9)	28 (2.3)	
Neither low nor high willingness	488 (13.5)	50 (7.8)	104 (18.8)	128 (19.4)	46 (8.0)	160 (13.4)	
High willingness	3054 (84.2)	584 (91.2)	423 (76.4)	519 (78.8)	520 (90.1)	1008 (84.3)	

Percentages were calculated from the number of respondents to each question

* The willingness to take on more responsibility was not investigated among doctors, as it is assumed that they already bear a great deal of responsibility

### Associations between willingness to take on or relinquish more responsibility and to strengthen interprofessional collaboration

Between 2482 and 2899 observations were included in the three models of the regression analyses regarding willingness to take on more responsibility (Table 3). The predictors of the extended model explain 47.7% of variance in the outcome variable. The regression results indicate that there is a statistically significant relationship between high willingness to take on more responsibility and the professionals’ willingness to strengthen IPC. Participants with high willingness to take on more responsibility had 5.30 times the odds of high willingness to strengthen IPC (95% CI 3.08–9.11, *p* < .001) compared to those with low willingness to take on more responsibility.

The sensitivity analysis (Supplementary Table S2, Additional File 2) also shows a similar direction in the relationship, despite a change in the categorization of the variable on willingness to take on more responsibility. However, the sensitivity analysis also makes clear that the willingness to strengthen IPC of the participants differs depending on the degree of their willingness to take on more responsibility.

**Table 3:** OR and CI for willingness to strengthen IPC by willingness to take on more responsibility

	High willingness to strengthen interprofessional collaboration					
	Crude analysis		Basic model^a^		Extended model^b^	
Predictors	OR (95% CI)	*P*-value	OR (95% CI)	*P*-value	OR (95% CI)	*P*-value
**Willingness to take on more responsibility**: Reference = Low willingness						
Neither low nor high willingness	1.55 (0.95–2.49)	0.073	1.59 (0.97–2.56)	0.059	1.64 (0.92–2.91)	0.093
High willingness	5.21 (3.28–8.14)	< 0.001	5.54 (3.48–8.69)	< 0.001	5.30 (3.08–9.11)	< 0.001
Observations	2899		2888		2482	
R^2^ Nagelkerke	0.180		0.191		0.477	

^a^ adjusted for age and sex

^b^ adjusted for age, sex, profession, professional experience, type of employment, and region

In the three models of the regression analyses regarding willingness to relinquish more responsibility, between 2900 and 3341 observations were included (Table 4). The predictors of the extended model explain 47% of variance in the outcome variable. The regression results indicate that there are statistically significant relationships between high willingness to relinquish more responsibility and the professionals’ willingness to strengthen IPC. Participants with high willingness to relinquish more responsibility had 3.30 times the odds of high willingness to strengthen IPC (95% CI 2.40–4.54, *p* < .001) compared to those with low willingness to relinquish more responsibility. Those who indicated a neither low nor high willingness to relinquish more responsibility had 1.57 time the odds of high willingness to strengthen IPC (95% CI 1.17–2.10, *p* < .01). The effect sizes show that the relationships between high willingness to relinquish more responsibility and the professionals’ willingness to strengthen IPC is weaker than the relationship between high willingness to take on more responsibility and the professionals’ willingness to strengthen IPC.

The consistency of our findings becomes evident through our sensitivity analysis (Supplementary Table S3, Additional File 3). Despite the modification made to the categorization of the variable regarding willingness to relinquish more responsibility, the odds ratios and significance levels remained largely consistent.

**Table 4:** OR and CI (95%) for willingness to strengthen IPC by willingness to relinquish more responsibility

	High willingness to strengthen interprofessional collaboration					
	Crude analysis		Basic model^a^		Extended model^b^	
Predictors	OR (95% CI)	*P*-value	OR (95% CI)	*P*-value	OR (95% CI)	*P*-value
**Willingness to relinquish more responsibility**: Reference = Low willingness						
Neither low nor high willingness	1.47 (1.14–1.89)	0.002	1.48 (1.15–1.91)	0.002	1.57 (1.17–2.10)	0.003
High willingness	2.69 (2.05–3.52)	< 0.001	2.81 (2.13–3.69)	< 0.001	3.30 (2.40–4.54)	< 0.001
Observations	3341		3325		2900	
R^2^ Nagelkerke	0.184		0.210		0.470	

^a^ adjusted for age and sex

^b^ adjusted for age, sex, profession, professional experience, type of employment, and region

## Discussion

We found that members of all allied health professions were highly willing to take on more responsibility for decision-making in patient care. Medical practice assistants and nurses were highly willing to relinquish responsibility, but pharmacists and physiotherapists less so; doctors were torn between high and neither high nor low willingness. Members of all professions were highly willing to strengthen IPC. We found a strong, statistically significant relationship between willingness to take on more responsibility and willingness to strengthen IPC. The relationship between willingness to relinquish responsibility and willingness to strengthen IPC was smaller.

Based on our findings, the five largest professional groups in healthcare in Switzerland partly fulfil the two identified prerequisites to overcoming barriers to implement IPC. Firstly, they are willing to strengthen IPC, increasing the chance of initiatives driven by policymakers or associations to yield substantial advancements in IPC. Secondly, allied health professionals are willing to take on more responsibility and some are also open to handing over responsibility to others. However, not all doctors are willing to relinquish and share responsibility with allied health professionals.

Our results are in line with previous studies which found health professionals to agree on the importance of IPC, emphasising their willingness to collaborate [37] and their optimistic attitude towards IPC [16, 31, 58]. In an analysis based on Ajzen’s theory of planned behaviour, Zielińska-Tomczak et al. [31] similarly found that openness to collaboration between doctors and pharmacists could encourage their intention to establish a professional partnership. Doctors and pharmacists were open to collaboration. However, their subjective norms and perceived behavioural control could reduce their willingness to collaborate. In their study, the researchers found that doctors and pharmacists had concerns regarding the limited understanding of pharmacists‘ skills and doctors’ mistrust of pharmacists’ intentions [31]. The differences we found between allied health professionals and doctors confirm the results of previous studies which show significantly different attitudes of health professionals towards interprofessional collaboration [7]. These studies found that physiotherapists had the most positive attitude compared to many professional groups [58], pharmacists showed a favourability towards establishing collaboration [31], and nurses demonstrated a more favourable attitude than doctors [7, 34, 59].

The differences between professional groups in their willingness to strengthen IPC have also been described in previous studies. Haddara and Lingard [60] found that there are two major discourses in IPC: the utilitarian and the emancipatory discourse. While doctors tend to follow the utilitarian discourse and focus on the value of IPC to improve patient outcomes, allied health professionals tend to follow the emancipatory discourse. For them, IPC is primarily about equalising power relations among health professionals. This theory can potentially be used to explain our results. Accordingly, pharmacists, nurses and physiotherapists in Switzerland might support efforts for more IPC in order to gain more respect for their work and more autonomy of doctors [6].

The ambivalence of doctors regarding their willingness to relinquish responsibility and their comparatively lower willingness to strengthen IPC could be explained primarily by the findings of Baker et al. [6] and Whitehead [17] and reflect the doctor dilemma that is also evident in our data. Many doctors may fear that IPC poses a potential threat to their professional status, including their dominant position in the division of labour of the health professions with significantly more autonomy, higher income and more prestige [6]. Furthermore, they might have difficulties in relinquishing responsibility for decision-making and engaging in interprofessional collaboration because of their professional socialization and education as leaders and decision-makers, as also described by Aase et al. [25] and van Duin et al. [22]. Nevertheless, many doctors already fulfil the prerequisite for the implementation of IPC because they are willing to relinquish responsibility and follow the instruction of IPE programs to share responsibility with other health professionals.

We are unsure to what extent health professionals taking on more responsibility actually increases interprofessional activities. On the one hand, based on the arguments of Baker et al. [6] and Whitehead [17], it is conceivable that increasing responsibility does not lead to improved IPC, because these efforts do not bring any additional benefit to the professionals. The ‘doctor dilemma’ might therefore not be a ‘doctor’ but a ‘health professionals’ dilemma’. On the other hand, Rawlinson et al. [15] showed that many organizational barriers make IPC more difficult. Allied health professionals have a larger scope for IPC in their daily work compared to doctors. They can often allow themselves more time, are more flexible in their management of workload and are often financed by other, service-independent remuneration models. Therefore, allied health professionals may have a higher chance to successfully engage in interprofessional collaboration.

The differences between the health professional groups regarding the willingness to relinquish responsibility raise exciting questions. Besides the explanations regarding doctors’ hesitation to relinquish responsibility in decision-making because of their professional socialization [6, 9, 15, 17, 22], we did not find further literature on this matter. In view of health professional education and the actual activities in practice, it is conceivable that doctors, medical practice assistants and nurses often have to take on activities in their daily work that do not belong to their task and competence profile but for which there are no other available staff or for which it would be more time-consuming to involve the appropriate professional than to take on the task themselves (e.g. making appointments, discussions with insurers or writing reports). Thus, a larger part of their work might involve tasks that do not correspond to their learned competences and their interests. This could explain why medical practice assistants, nurses and many doctors were willing to relinquish responsibility in decision-making for the care of their patients. For pharmacists and physiotherapists, on the other hand, it is conceivable that their daily work already corresponds better to their learned competences and that they see fewer meaningful opportunities to delegate tasks to other professionals.

### Implications for policy, research, and practice

Our findings show that there is a great untapped potential in the willingness of health professionals to strengthen IPC. Based on our findings, to further reinforce IPC in practice, endeavours to increase the professionals’ willingness to take on more responsibility and to distribute responsibility in decision-making in patient care among the health professionals involved seems promising. Increasing their willingness to relinquish responsibility might also be important but its connection to IPC is weaker.

Policymakers should consider a change of the regulations that limit the translation of the health professionals’ willingness into practice as allied professions are currently not allowed to take on more responsibility for first contact and coordination of care.

In terms of research, a longitudinal study is needed to study the actual acceptance and relinquishment of responsibility and changes in interprofessional activities of health professionals.

A starting point in practice could be to assign certain health professionals the responsibility for decision-making in the provision of care for specific patient groups. This could be encouraged by adapting job descriptions and referral processes for patients within practices and between healthcare providers or other health professionals. Defining patient pathways and providing secure communication tools and electronic data exchange to support care and coordination with other professionals is important to enable shared responsibility.

To enable health professionals to take on or relinquish responsibility and to decide when it is appropriate to take on or relinquish responsibility, Whitehead’s recommended efforts [17] in their education and training are necessary. These include the development of training programs which focus on specific skills that have been shown to improve patient outcomes to engage doctors in models of team care [17]. Moreover, the formal and hidden curricula in medical education should be examined to find ways to train medical students both to accept and share responsibility [17]. Another approach that could be taken to enable allied health professionals to take on more responsibility is to introduce ‘entrustable professional activities’ (EPAs) as a framework for practice-based training and assessment in the undergraduate training of allied health professionals. A general definition of an EPA is ‘a unit of professional practice that can be fully entrusted to a trainee, once he or she has demonstrated the necessary competence to execute this activity unsupervised‘ [61]. One argument for defining and implementing EPAs in allied health professional education could be to clarify which professional skills and competences must be specifically trained during education and mastered with a certain level of supervision so that responsibility for these activities can be assumed [62].

### Study strengths and limitations

This is the first study measuring the association between health professionals’ willingness to take on or relinquish responsibility in decision-making for the care of their patients and their willingness to strengthen IPC. Moreover, this research is relevant in addressing persisting problems in IPC in healthcare in Switzerland.

The successful execution of our survey, encompassing health professionals with different professional backgrounds and sociodemographic characteristics, was made possible through the collaboration with many public and private stakeholders in Swiss healthcare. This collaborative effort led to a large sample size for our study. We made extensive efforts to promote the dissemination of our survey and motivate as many health professionals as possible to participate in our study, thereby striving to implement the principle of probability sampling.

While our sample closely aligns with the target population in terms of sex and age distribution, it is important to acknowledge the potential for selection bias within our data [63]. Given our study’s reliance on self-reports, the prospect of random measurement errors and self-report bias must also be acknowledged [64].

We implemented several procedural controls to mitigate potential social desirability bias or common method variance in our study in line with the recommendations of Converse et al. [65] and Peabody et al. [66]. Specifically, to minimize respondents’ perception of a ‘desired’ answer, participants were assured of the anonymity of their responses, thereby reducing the risk of socially desirable responding. Following Kock et al. [67] independent and dependent variables were measured using three distinct questions at different stages of the survey, which comprised a wide range of topics. This approach helped to prevent consistency bias and ensured that responses were not influenced by transient impressions or attitudes. Furthermore, we framed the questions to assess participants’ willingness in their current professional position, rather than in a general context, to focus responses on their real-life context rather than an idealised, socially desirable professional setting. Despite the procedures we have implemented, we acknowledge the potential for overestimating the actual willingness to take on or relinquish responsibility and the willingness to strengthening IPC [65, 67, 68].

Similar to all cross-sectional investigations [69], our study encounters the constraint of evaluating both exposure and outcome concurrently at a single point in time. Our study did not aim to, nor can it, establish a causal relationship between health professionals’ willingness to take on or relinquish responsibility and their willingness to strengthen IPC.

Despite our provision of a definition of IPC and professional responsibility for decision-making for the care of patients, we cannot exclude the possibility that the participants had differing understandings of both terms. The findings by Haddara and Lingard [60] on different discourses and understandings of IPC provide evidence of this potential limitation that could have affected our data. In addition, our survey was only accessible in both German and French, potentially leading to underrepresentation from the Italian-speaking canton of Ticino. Consequently, the generalizability of our findings to the Italian-speaking region of Switzerland is limited.

## Conclusion

Increasing the health professionals’ willingness to take on responsibility is crucial to strengthen IPC. Increasing the willingness to relinquish responsibility would likely be less effective. Actions required include educational and political efforts to transfer responsibility to allied health professionals and to enable health professionals to decide when it is appropriate to take on or relinquish responsibility. Given the willingness of many health professionals to strengthen IPC, substantial potential in practice is evident.

## Electronic supplementary material

Below is the link to the electronic supplementary material.

Supplementary Material 1

Supplementary Material 2

Supplementary Material 3

## Data Availability

The datasets used and analysed during the current study are available from the corresponding author on reasonable request.

## Abbreviations

IPC: Interprofessional collaboration
IPE: Interprofessional education
OR: Odds ratio
CI: Confidence interval
SD: Standard deviation
