# Supplementary material for: Health professionals’ willingness to share responsibility and strengthen interprofessional collaboration: a cross-sectional survey
Source: BMC Med Educ. 2025 Jan 21;25:102. doi: 10.1186/s12909-024-06351-9 (PMC11753034; doi:10.1186/s12909-024-06351-9)
Supplement: Supplementary file 1 — Supplementary Material 1 [file 12909_2024_6351_MOESM1_ESM.pdf]

Supplementary information

Additional file 1

Supplementary Table S1: Questionnaire and variables from survey «Health 2040»

Variable	Question	Response options	Used in analysis
**Sociodemographic characteristics**			
Sex	Please indicate your sex.	o Female<br>o Male<br>o Diverse	Independent variable
Age	What is your year of birth?	Dropdown menu	Independent variable
Professional background	Which of the following statements apply to you?<br>Patients always include clients.	o I work in a medical or health profession with contact to patients.<br>o I work in a medical or health profession without contact with patients.<br>o I do not practise a medical or	

Variable	Question	Response options	Used in analysis
		health profession.	
Profession	To which professional group do you currently belong?	○ Pharmacists<br>○ Physicians<br>○ Medical practice assistants<br>○ Nurses<br>○ Physiotherapists<br>○ Other profession in the health sector, please specify [text field].	Independent variable
Professional experience (overall)	How many years of professional experience do you have in the health sector?	Dropdown menu	Independent variable
Type of employment		○ Employed with management responsibility<br>○ Employed without management responsibility<br>○ Self-employed	Independent variable

Variable	Question	Response options	Used in analysis
		with employees<br>○ Self-employed without employees	
Region of work		○ Urban<br>○ Intermediate<br>○ Rural	Independent variable
**Willingness**			
Willingness to take on more responsibility	In your current position, how willing are you to take on more professional responsibility in decision-making for the care of your patients?	○ Very low<br>○ Low<br>○ Neither low nor high<br>○ High<br>○ Very high	Independent variable
Willingness to relinquish more responsibility	In your current position, how willing are you to relinquish more professional responsibility in decision-making for the care of your patients?	○ Very low<br>○ Low<br>○ Neither low nor high<br>○ High<br>○ Very high	Independent variable
Willingness to strengthen interprofessional	In your current position, how willing are you to strengthen	○ Very low<br>○ Low	Dependent variable

Variable	Question	Response options	Used in analysis
collaboration	interprofessional collaboration?	○ Neither low nor high<br>○ High<br>○ Very high	

5 www.health2040.ch
