# Supplementary material for: Health professionals’ willingness to share responsibility and strengthen interprofessional collaboration: a cross-sectional survey
Source: BMC Med Educ. 2025 Jan 21;25:102. doi: 10.1186/s12909-024-06351-9 (PMC11753034; doi:10.1186/s12909-024-06351-9)
Supplement: Supplementary file 2 — Supplementary Material 2 [file 12909_2024_6351_MOESM2_ESM.pdf]

Supplementary information

Additional file 2

Supplementary Table S2: OR and CI for willingness to strengthen IPC by willingness to take on more responsibility

	**High willingness to strengthen interprofessional collaboration**					
	Crude analysis		Basic model[a]		Extended model[b]	
Predictors	OR (95% CI)	*P*-value	OR (95% CI)	*P*-value	OR (95% CI)	*P*-value
**Willingness to take on more responsibility:** Reference = Low willingness						
High willingness	3.62 (2.89 – 4.55)	<0.001	3.77 (2.99 – 4.74)	<0.001	3.52 (2.68 – 4.64)	<0.001
Observations	2899		2888		2482	
R^2 Nagelkerke	0.179		0.190		0.476	

[a] adjusted for age and sex

[b] adjusted for age, sex, profession, professional experience, type of employment, and region

OR: Odds ratio, CI: Confidence interval, IPC: Interprofessional collaboration
