# Supplementary material for: Health professionals’ willingness to share responsibility and strengthen interprofessional collaboration: a cross-sectional survey
Source: BMC Med Educ. 2025 Jan 21;25:102. doi: 10.1186/s12909-024-06351-9 (PMC11753034; doi:10.1186/s12909-024-06351-9)
Supplement: Supplementary file 3 — Supplementary Material 3 [file 12909_2024_6351_MOESM3_ESM.pdf]

Supplementary information

Additional file 3

Supplementary Table S3: OR and CI for willingness to strengthen IPC by willingness to relinquish more responsibility

	**High willingness to strengthen interprofessional collaboration**					
	Crude analysis		Basic model[a]		Extended model[b]	
Predictors	OR (95% CI)	*P*-value	OR (95% CI)	*P*-value	OR (95% CI)	*P*-value
**Willingness to relinquish more responsibility:** Reference = Low willingness						
High willingness	2.03 (1.66 – 2.50)	<0.001	2.11 (1.72 – 2.61)	<0.001	2.37 (1.87 – 3.01)	<0.001
Observations	3341		3325		2900	
R^2 Nagelkerke	0.180		0.206		0.467	

[a] adjusted for age and sex

[b] adjusted for age, sex, profession, professional experience, type of employment, and region

OR: Odds ratio, CI: Confidence interval, IPC: Interprofessional collaboration
